# Innate Lymphoid Cells (ILCs) as Mediators of Inflammation, Release of Cytokines and Lytic Molecules

**DOI:** 10.3390/toxins9120398

**Published:** 2017-12-10

**Authors:** Noha Mousaad Elemam, Suad Hannawi, Azzam A. Maghazachi

**Affiliations:** 1 Department of Clinical Sciences, College of Medicine, and Sharjah Institute for Medical Research (SIMR), University of Sharjah, Sharjah 27272, UAE; noha.elemam211@gmail.com; 2 Medical Department, Ministry of Health and Prevention, Dubai 65522, UAE; suad1@ausdoctors.net

**Keywords:** NK cells, innate lymphoid cells, lytic molecules, inflammation

## Abstract

Innate lymphoid cells (ILCs) are an emerging group of immune cells that provide the first line of defense against various pathogens as well as contributing to tissue repair and inflammation. ILCs have been classically divided into three subgroups based on their cytokine secretion and transcription factor profiles. ILC nomenclature is analogous to that of T helper cells. Group 1 ILCs composed of natural killer (NK) cells as well as IFN-γ secreting ILC1s. ILC2s have the capability to produce T_H_2 cytokines while ILC3s and lymphoid tissue inducer (LTis) are subsets of cells that are able to secrete IL-17 and/or IL-22. A recent subset of ILC known as ILC4 was discovered, and the cells of this subset were designated as NK17/NK1 due to their release of IL-17 and IFN-γ. In this review, we sought to explain the subclasses of ILCs and their roles as mediators of lytic enzymes and inflammation.

## 1. Introduction

Innate lymphoid cells (ILCs) are crucial effectors of innate immunity as they are the primary line of defense against different types of pathogens, in addition to their vital contribution in tissue repair and asthma [1]. ILCs have the ability to interact with a wide variety of hematopoietic and non-hematopoietic cells to coordinate immunity and homeostasis in multiple human tissues. ILCs act as bodyguards at the barrier surfaces, which are common sites for pathogen invasion, where they provide immunity against viruses, bacteria, and parasites. These cells are able to detect changes in the microenvironment and hence, produce cytokines that constrain the damages caused by pathogens or modulate the immune response. 

ILCs are a family of immune cells that are defined by several features including the absence of recombination activating gene (RAG)-dependent rearranged antigen receptors, their lymphoid morphology as well as lack of myeloid phenotypic markers, and are therefore called cell lineage marker-negative (Lin^−^) cells [2,3,4,5,6]. ILCs are classified according to their transcription factors and cytokine production profile that to a large extent parallels that of T helper (T_H_) cell subsets. The cells have been grouped into cytotoxic ILCs and helper ILCs mirroring CD8^+^ T cytotoxic cells and CD4^+^ T helper cells, respectively [7,8]. Natural killer (NK) cells represent the cytotoxic ILC population [9], while helper ILC populations are further subdivided into three groups [7,10]. Accordingly, group 1 comprises ILC1s and NK cells representing type 1 immune response, group 2 includes ILC2s that produce type 2 cytokines such as IL-5 and IL-13, and group 3 includes ILC3s and lymphoid tissue inducers (LTis) that produce IL-17 and/or IL-22 analogous to T_H_17/T_H_22 cells [11]. The ILC4 group comprises cells that release IL-17 and IFN-γ, also known as NK17/NK1 cells [12].

ILCs are mediators of inflammation in various organs such as the intestine, respiratory system as well as in tissue remodeling and repair in the skin. The recruitment, activation, and action of ILCs are controlled by cytokines and growth factors that are selective for each ILC group. Moreover, ILCs, and especially ILC3s, have key roles in restraining tissue resident commensal bacteria [13]. In this review, a brief overview of each ILC group is described along with their roles in inflammation and toxicity of different organs.

### Development of Innate Lymphoid Cells 

Lymphocytes usually emerge from common lymphoid progenitor (CLP) cells that differentiate into precursors, which further commit to a particular cell lineage [14,15,16]. The different subsets of the ILC family have interconnected developmental pathways and share an ancestry, distinct from the one that develops into T and B cells. The precursor cells express the integrin α4β7 and are referred to as α-lymphoid precursor (αLP) cells, which also express chemokine receptor CXCR6. These αLP cells include the common helper ILC and NK cell precursors. Furthermore, the transcriptional repressor Id2 (inhibitor of DNA-binding protein) is expressed by these precursors which can develop into ILCs and/or NK cells [15,17,18], but inhibit the expansion of B and T cells [19]. ILCs are similar to T cells in their development as both require functional IL-7 receptor signaling, where IL-2 receptor common γ chain and Janus kinase 3-deficient patients lack T cells and ILCs due to the absence of IL-7R signaling.

One of the Id2^+^ ILC precursors expressed CD127 (IL-7Rα), and is distinguished by the lack of FLT3 (FMS-related tyrosine kinase 3) and CD93 [15]. Other studies identified an ILC precursor by the expression of the transcription factor promyeloid leukemia zinc finger (PLZF; encoded by Zbtb16), that is necessary for the development of NKT cells [20,21]. These cells were reported to express CD127, α4β7, Thy1, and CD117 (c-Kit) and were found in fetal liver and adult bone marrow in mice. They gave rise to CD127^+^ ILC1s, ILC2s, and ILC3s but were unable to develop into conventional NK cells or LTi cells [18]. Additionally, all human ILC populations, including cytotoxic NK cells, can be generated in vitro from a common CD34^+^ ILC progenitor expressing RORγ t, CD34, CD45RA, CD117, CD161, integrin α4β7, and high levels of IL-1 receptor type 1 (IL1R1). This precursor did not give rise to T cells but generated ILC1s, ILC2s, and ILC3s, as well as NK cells, hence, representing a more restricted ILC precursor [22]. Complete understanding of the development of ILCs starting from CLPs towards each ILC subset has not yet been accomplished and thus requires further experimental work in both mice and humans. 

## 2. Classification of Innate Lymphoid Cells

### 2.1. ILC1s Group

Group 1 ILCs comprises cells that have the ability to produce type 1 inflammatory cytokines, particularly IFN-γ and tumor necrosis factor (TNF-α), but are unable to produce T_H_2 and T_H_17 cell-associated cytokines. The classical members of this group are NK cells that were first described in 1975 as innate effector cells that display cytotoxic activity towards tumor cells [23,24]. NK cells are lymphocytes that bridge the innate and adaptive immune systems and respond to tumors and infected cells. NK cells circulate in the peripheral blood, and rapidly migrate into sites of immune reactions in peripheral tissues such as secondary lymphoid organs, via gradients of cytokines and chemokines [25]. It is well recognized that NK cells function is not only cytotoxicity but also production of inflammatory molecules and various lytic enzymes upon activation. These cells are developed in the bone marrow, but can also develop in the thymus and hence, these two types of NK cell populations may have divergent requirements for their development [26]. The T_H_1 cell associated transcription factor T-bet (which is encoded by T-box transcription factor-Tbx21) cooperates with eomesodermin (Eomes), another T-box transcription factor, in order to control the development and function of NK cells [26]. 

NK cell function is tightly regulated by a repertoire of membrane-expressed inhibitory and activating receptors, which are the “nuts and bolts” of NK cell function, respectively [27,28]. NK cell inhibitory receptors include members of the C-type lectin-like receptor family such as the NKG2A/CD94 heterodimer [29,30]. Additionally, killer immunoglobulin-like receptors (KIRs) play a crucial role in inhibiting NK cells as they interact with highly polymorphic MHC class I molecules [31]. Activating receptors allow the recognition of altered self-antigens that are expressed on stressed cells which act as danger signals. However, activation of NK cells occurs when a critical threshold of activating signal exceeds that of inhibition. The major activating receptors include NKG2D as well as several NCRs such as NKp30, NKp44, and NKp46, which are involved in the clearance of both tumor and virally infected cells [32,33]. 

The classical way of classifying human NK cells is based on the intensity of the expression of CD56 and CD16 molecules, where several studies have reported that CD56^dim^/CD16^+^ cells predominantly mediate cytotoxicity, whereas CD56^bright^ cells appear to principally secrete cytokines and in particular IFN-γ [34,35,36]. Furthermore, CD56^dim^ cells represent the major population, whereas CD56^bright^ cells represent around 10–15% of circulating NK cells, but are predominantly found in normal tissues and secondary lymphoid organs, such as lymph nodes and tonsils, and hence respond to locally produced cytokines [37,38]. 

Other studies suggest different systems for NK cell classification. For example, human NK cells incubated with IL-12 produced T_H_1-like cytokines such as IFN-γ and were termed NK1 cells, while those incubated with IL-4 produced T_H_2-like cytokines which include IL-5 and IL-13 and were termed NK2 cells [39]. It was also demonstrated that NK cells could be divided into subsets based on the expression of chemokine receptors. In this classification, primary NK cells express the CXC-chemokine receptor (CXCR1, CXCR3, and CXCR4), while other subsets express the CC-chemokine receptor (CCR1, CCR4, CCR5, CCR6, CCR7, and CCR9), as well as CXCR5 and CXCR6 within both CD56^bright^ and CD56^dim^ subsets [40]. The differential chemokine receptor expression profile would allow discrete, unique trafficking programs to exist, which likely influence NK cell function in innate and adaptive immunity. Therefore, NK cells could be divided into sub-categories according to their release of inflammatory cytokines and chemokines, as well as their differential expression of chemokine receptors [25]. 

**Figure 1:**
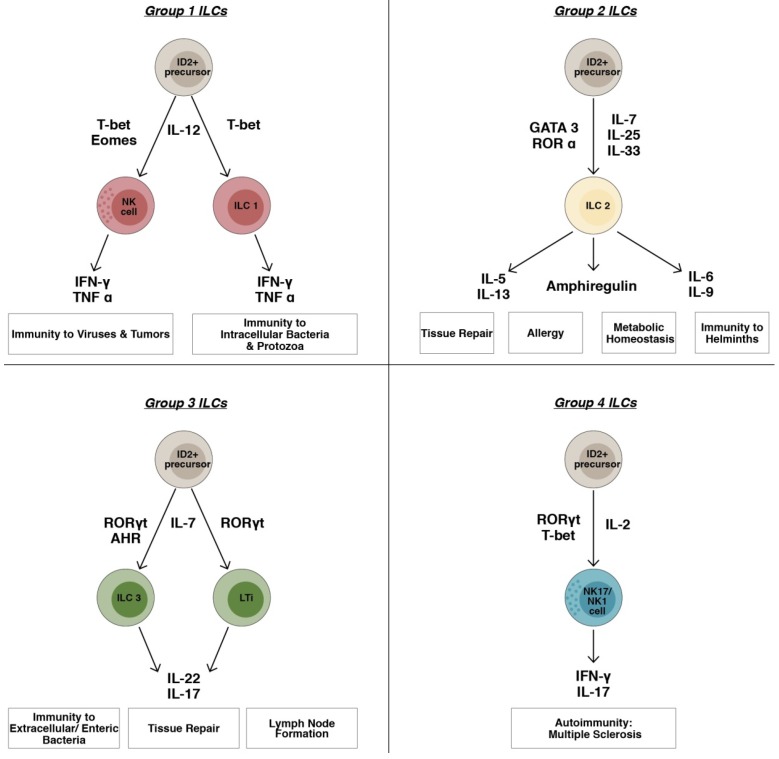
Classification of ILCs into four groups on the basis of transcription factors and cytokine profiles. ILCs were previously classified into three groups according to the differential expression of transcription factors and cytokine production profiles. The fourth group of ILCs comprises NK17/NK1 cells that are abundant in the CSF of multiple sclerosis patients. Abbreviations: AHR: aryl hydrocarbon receptor, Eomes: eomesodermin, GATA3: GATA-binding protein 3, IFN-γ: Interferon-gamma, IL: Interleukin, ILC: Innate lymphoid cell, NK: Natural Killer Cell, ROR: Retinoic acid receptor-related orphan receptor, T-bet: T-box transcription factor-Tbx21, TNF α: Tumor necrosis factor alpha.

As shown in Figure 1, other IFN-γ-secreting ILCs have been described and are referred to as ILC1s that are distinct from NK cells [41]. These ILC1s have been identified in mice and humans, and are able to produce IFN-γ but not any of the T_H_2 or T_H_17 cell-associated cytokines [42]. In humans, ILC1 subset expresses high levels of T-bet, and to a moderate level RORγt, but lacks the expression of CD117 and Eomes transcription factor [9]. These ILC1s are found in both mouse and human intestine and lung tissues, where they participate in type 1 inflammation [15,43]. Several studies have pointed out the pathogenic roles of ILC1s in the development of intestinal inflammation. For instance, the population of IFN-γ producing ILC1s was expanded in human inflammatory bowel (IBD) disease and specifically in inflamed intestinal tissues of Crohn’s disease patients. [44,45]. Of note, the intraepithelial ILC1 is a population of CD127^+^ cells present in the gastrointestinal epithelia and tonsils [46]. Besides, ILC1s contribute to immune responses against intracellular pathogens, such as Toxoplasma gondii and Clostridium difficile [15,47]. 

### 2.2. ILC2s Group

An innate immune cell that possesses the capacity to secrete type 2 cytokines was postulated after IL-25 intranasal administration thus triggering the production of IL-5 and IL-13 in RAG-2^−/−^ mice which lack B and T cells [48,49,50]. Group 2 ILCs development requires IL-7 in order to produce T_H_2 cell-associated cytokines along with stimulation with IL-25 and IL-33 [48,49,51]. Several studies characterized these type 2 cytokine-producing ILCs and reported their presence in the mesenteric fat-associated lymphoid clusters, mesenteric lymph nodes, liver, intestine, and spleen. The role of ILC2 cells in the pathogenesis of asthma and their orchestration of T_H_2 immunity has been previously reviewed [1].

Several studies characterized type 2 cytokine-producing ILCs and reported their presence in the mesenteric fat-associated lymphoid clusters, mesenteric lymph nodes, liver, intestine, and spleen. The first study conducted on human ILC2s indicated a subset of cells that possess the phenotype Lin^−^ IL-7Rα^+^ CD45^hi^. ILC2s population was reported to express transcripts encoding IL-13, IL-17 receptor B (IL-17RB; a component of the IL-25 receptor), ST2 (a component of the IL-33 receptor), CRTH2 (chemoattractant receptor-homologous molecule prostaglandin D2 receptor), and CD161. Moreover, ILC2s require the transcription factors GATA-binding protein 3 (GATA3), and retinoic acid receptor-related orphan receptor-α (RORα) [52,53,54]. 

As illustrated in Figure 1, ILC2s have been reported to produce pro-inflammatory cytokines such as GM-CSF, IL-3, IL-6, IL-8, IL-9, and IL-21 [8,55]. Apart from cytokines, lipid mediators regulate human ILC2 function, where prostaglandin D2 (PGD2) was shown to activate ILC2 cytokine secretion and migration, and lipoxin A4 inhibits ILC2 function [56,57]. In addition to their characteristic surface markers, ILC2s possess chemokine receptors, such as CCR9, CXCR4, and CXCR6, which are involved in the homeostatic distribution of lymphoid cells [58]. 

ILC2 cells are present in the skin, adipose tissues, as well as tonsils [59,60]. Host protection against parasites requires type 2 responses, where IL-4, IL-5, IL-9, and IL-13 were reported to increase mucus production and smooth muscle contractility, promote goblet cell hyperplasia, and control the activation of macrophages and granulocytes thus participating in the control of parasitic infections [61,62,63,64]. In mice, lung ILC2s contribute to the immune response against nematode, viruses and fungi [65]. Other studies showed that ILC2s play an important role in metabolic homeostasis, where ILC2 responses limit high-fat diet induced obesity and insulin resistance [66,67]. 

Human ILC2s exhibit contradictory roles in the inflammation of the respiratory tissues [1,68,69]. It was reported that ILC2 are involved in lung homeostasis and repair of damaged respiratory tissues [68,70]. This could be due to their production of the epidermal growth factor-related amphiregulin repairing airway epithelial cell evoked by pathogenic viruses, which is in line with the finding that ILC2s have a crucial role in tissue repair and wound healing [69,71]. On the contrary, human ILC2s are robustly expanded in other type 2 inflammatory diseases such as chronic rhinosinusitis (CRS), as they were found in the peripheral blood and lungs with increased frequency in the nasal polyps of patients with CRS [8]. Likewise, ILC2 cells produce IL-5 which activates eosinophils that are also abundantly present in nasal polyps of patients with CRS [72]. Additionally, ILC2s could contribute to allergic lung inflammation. Chang et al. showed an exacerbation of allergic lung inflammation by ILC2s in a mouse model of virus-mediated allergic asthma [70]. ILC2s could also contribute to allergic lung inflammation coordinating with DCs and CD4^+^ T cells at mucosal sites [73,74]. 

Human ILC2s might also be involved in allergic asthma as higher proportions of these cells are present in airway tissues, including bronchoalveolar lavage fluid and sputum, as well as PBMCs of asthmatic patients, where they produce large amounts of IL-5 and IL-13 upon activation with IL-25 and IL-33 [75,76,77,78]. Their contribution was further illustrated where ILC2s were found to be increased in the peripheral blood of asthmatic people relative to the controls [75]. Another study reported that ILC2s capable of secreting IL-5 and IL-13 are increased in the sputum and lung airways upon allergen induction in asthmatic patients [79]. The involvement of ILC2 cytokines such as IL-5 and IL-13 in the respiratory inflammation is quite extensive and thus, monoclonal antibodies targeting them are in clinical trials for the treatment of refractory asthma and CRS [80]. 

In the skin, the ILC2 population was found to be expanded in the lesions of patients with atopic dermatitis, an inflammatory skin condition characterized by elevated levels of IL-5, IL-13, IL-25, IL-33, TSLP, and PGD2 [81,82,83,84]. Similarly, ILC2s were enriched in patients with house dust mite allergy [84,85]. In the intestine, ulcerative colitis (UC) is characterized by type 2 inflammation with IL-4, IL-5, and IL-13 cytokines as key pathological players. This was further illustrated by a study where these cytokines levels were associated with the severity of the disease [86,87].

### 2.3. ILC3s Group

Similar to T_H_17 cells, group 3 ILCs is defined by the ability of the cells to produce the cytokines IL-17A with or without IL-22. As illustrated in Figure 1, ILC3s depend on the transcription factor RORγt as well as IL-7Rα for their development and function. Both murine and human ILC3 are identified as Lin^−^ CD127^+^ RORγ t^+^ cells. ILC3s include two major cell subsets; fetal LTis and postnatal ILC3s. The classical members of group 3 ILCs are LTi cells, which are crucial for the formation of secondary lymphoid organs including peripheral and mesenteric lymph nodes, Peyer patches, colonic patches, and cryptopatches [88]. LTi cells are among the first cells to populate the lymph node and are recognized as vital regulators of lymphoid tissue architecture after birth. Several studies pointed out the presence of lymphoid cells present in the intestine that express NKp46 but do not resemble NK cells [89,90]. These NKp46^+^ cells possess the transcription factor RORγt, lack cytotoxic effectors such as perforin, granzymes and do not produce IFN-γ or TNF, but instead produce the cytokine IL-22. They are called NCR^+^ ILC3s and are distinct from LTi cells that secrete IL-22 and hence, are also known as NK22 cells, NCR22 cells, or NKR-LTi cells [91,92]. NK22 cells also appeared in the small intestine lamina propria during bacterial infection, and could help in limiting inflammation and protecting the mucosal surface during infections [93]. 

ILC3s are divergent in both mice and humans. In humans, almost all ILC3s express CCR6 and CD117, and could be distinguished on the basis of NKp44 expression. Adult human ILC3s can be subdivided into NKp44^+^ and NKp44^−^ subsets. NKp44^+^ ILC3s are the most prevalent cells in the human gut under homeostatic conditions. These cells produce IL-22 and consequently, maintain gut barrier function in mice and are also likely to be tissue protective in the human gut [85]. On the other hand, NKp44^−^ ILC3s, in particular those expressing HLA-DR, produce limited amounts of IL-17 and thus are found to mediate colitis in mice. They are also enriched in the inflamed ileum and colon of patients with Crohn’s disease [90,94]. ILC3s have been identified after birth in many organs, including spleen, endometrium, decidua, skin, and lung [60,95,96,97]. Additionally, they are found in mucosal tissues, such as the small and large intestines, Peyer’s patches, and gut-associated lymphoid tissue. ILC3s present in human tonsils were found to produce GM-CSF, B-cell activating factor (BAFF), LIF and IL-22, and express high levels of CD40L and RANKL (receptor activator of nuclear factor kappa-B ligand).

ILC3s maintain the barriers between the intestinal epithelial and the immune system responses. IL-22-producing ILC3s play a critical role in tissue repair and especially in the regeneration of the inflamed intestine and radiation-damaged thymus [98,99] but on the other hand, lack of IL-22 induced intestinal inflammation as well as erosion of the epithelial membranes. Moreover, ILC3s were reported to be key players for the IL-22 mediated innate immune response against extracellular bacteria such as *Citrobacter rodentium* in the gut [85]. ILC3s might also participate in protecting immune response against enterobacteria *E. coli* strain O157/H7 that causes attaching effacing lesions in humans. However, sustained IL-22 and epithelial proliferation may promote tumorigenecity [100], where ILC3s have been identified to be present in high proportions in non-small cell lung cancer (NSCLC) tumor tissues [8], as well as colorectal cancer [101]. 

ILC3s represent a tissue-specific target in IBD as they are mediators of intestinal inflammation via cytokine production, lymphocyte recruitment, and reorganization of the inflammatory tissues [45,102]. This was shown by a reduction in the number of NKp44^+^ NKp46^−^ ILC3s in inflamed intestinal tissues of patients with Crohn’s disease [44,103]. On the other hand, IL-17 producing NKp44^−^ ILC3s have been found to be enriched in the inflamed ileum and colon of these patients [45]. 

Regarding inflammatory skin diseases, NKp44^+^ ILC3s, whether IL-17 or IL-22 producing cells have been associated with psoriasis vulgaris, as their numbers were increased in the blood and inflamed skin [46,60]. Therefore, targeting ILC3s can be a novel treatment strategy in patients with psoriasis. Additionally, there is an increased frequency of ILC3s in the peripheral blood of multiple sclerosis patients [104]. In the lung tissues, NKp44^−^ ILC3s represent the most abundant ILC group, despite the high frequency of ILC2s. In chronic obstructive pulmonary disease (COPD) patients, all groups of ILCs are involved and present in lung tissues. ILC1s and NKp44^−^ ILC3s populations were increased unlike ILC2s in lung tissues as well as in the peripheral blood [105,106]. In summary, ILC3s could produce IL-17A, IL-17F, IL-22, GM-CSF or TNF depending on the stimulus given. They may enhance antibacterial immunity, cause chronic inflammation, or induce tissue repair. 

### 2.4. ILC4s Group

A novel subset of human NK cells was reported to be CD56^+^ CCR4^+^ which express NK cell maturation markers and cytotoxicity receptors NKp30, NKp44, NKp46, as well as IL-2Rβ and γ. They were designated as “NK17/NK1” cells due to their ability to produce IL-17 and IFN-γ [12]. This nomenclature was based on T_H_ terminology as certain T cells secrete IFN-γ as well as IL-17 and are termed T_H_1/T_H_17 cells [107,108,109]. NK17/NK1 cells also express CCL22/MDC, the ligand for CCR4 which may contribute to the chemotactic migration of these and other cell types [110]. These cells were generated upon in vitro IL-2 activation of CD56^+^ cells from the blood of normal individuals or multiple sclerosis (MS) patients. Moreover, they are abundant in cerebrospinal fluid (CSF) of MS patients without any activation [12]. NK17/NK1 cells were reported to possess the transcription factors T-bet and RORγt, which are essential for the secretion of IFN-γ and IL-17, respectively. These cells are considered a discrete subset of NK cells due to their differential transcription factor expression profile. In addition, they possess the ability to lyse human myeloid leukemia K562 target cells. This cytolytic activity was potentiated by treating NK17/NK1 cells with different concentrations of vitamin D_3_, its analog calcipotriol, or FTY720 a drug for treating MS patients [110]. Hence, they could play a crucial role in lysing target cells under pathological conditions and during inflammation where IL-2 is released [111].

ILC4 (NK17/NK1) cells share common features among the three different ILC groups, albeit they do not exactly fit into any of the previously described groups. First, they express transcription factors T-bet and RORγt similar to ILC1 and ILC3 subsets, respectively, and are able to secrete IFN-γ and IL-17. Moreover, NK17/NK1 cells express NKp30, NKp44, and NKp46, analogous to most ILC1s and ILC3s. However, they do not express IL-7Rα (CD127), in contrast to helper ILCs.

In comparison with the role of ILCs, whether mounting a response to intra or extracellular pathogens, anti-helminthic, lymphoid tissue organogenesis, tissue repair or metabolic homeostasis, the role of ILC4s is not yet quite clear, as they were generated after IL-2 activation of NK cells from peripheral blood of healthy people or MS patients, besides their existence in CSF of MS patients without any prior activation (reviewed in [111]). A possible suggestion could be that they might be polarized to an inflammatory local microenvironment, such as the brain of MS patients [12]. Moreover, these cells were observed in the skins of psoriasis patients (A. A. Maghazachi, unpublished data). However, their exact role in other autoimmune diseases such as rheumatoid arthritis and type I diabetes, among others has not been fully elucidated. Therefore, the role of ILC4s in autoimmunity and inflammation should be further investigated. Figure 1 describes the classification of the four ILCs subsets and shows their roles in various inflammatory disorders.

## 3. ILCs as Mediators of Host-Derived Lytic Molecules

The various cytokines secreted by distinct ILC groups have been explored as protective molecules in multiple disorders. A study by Howitt et al. showed that parasites induced IL-25 release which then activates the production of IL-13 by ILC2s [112]. In addition to their IL-5 and IL-13 secretion, ILC2s also produce IL-6 and IL-9 [113], which have fundamental roles in anti-helminthic responses, such as resistance to the helminth *Nippostrongylus brasiliensis* [61]. Moreover, ILC2s produce the epidermal growth factor–related amphiregulin upon viral infection promoting airway epithelial cell repair [69,71]. 

Clostridium difficile infection (CDI), is a disorder where anaerobic bacteria invade the gut where natural flora is disrupted. In CDI, toxins as well as virulence factors are released disrupting the homeostasis of the intestinal epithelial barrier [114]. This epithelial-toxin interaction causes the activation of inflammatory immune cells at the site of infection. It has been reported that lack of ILC groups enhances the susceptibility to CDI [115]. Furthermore, ILCs were reported to have a crucial role in recovering from CDI [115] and specifically ILC1s and ILC3s due to their IFN-γ and IL-22 secretion, respectively [116]. Besides, ILC3s were found to regulate IL-17 secretion via the ligand-dependent transcription factor, aryl hydrocarbon receptor (AHR), whose ligands include environmental toxins such as dietary components as well as endogenous ligands [117].

ILC3s limit the propagation of commensal bacterial species and their toxic products in order not to reach the systemic immune system. One possible mechanism is via promoting the formation of isolated lymphoid follicles and epithelial cell fucosylation [11,89]. Another potential mechanism could be triggering ILC3s by microbiota to produce GM-CSF, promoting homeostasis with the help of DCs and T regulatory cells [118]. IL-22 induces rapid production of antimicrobial peptides such as α and β beta defensins by intestinal epithelial cells, as well as inducing epithelial cell survival and proliferation [119,120,121]. Moreover, ILC3-derived IL-22 protects against tissue damage occurring in intestinal epithelial stem cells due to graft versus host disease (GvHD), which occurs after hematopoietic stem cell transplantation [122]. This was illustrated by IL-22 treatment in mice that caused an intestinal stem cell survival improving epithelial cell renewal, and lessening of intestinal GvHD [10]. Therefore, IL-22-producing ILC3s from the recipient could be fundamental participants in limiting the damage during GvHD [122]. Furthermore, ILC3s play a crucial role in pregnancy, where NCR^+^ ILC3s produce CXCL8 and GM-CSF that induce the release of other cytokines by decidual neutrophils which promote tissue remodeling and maintenance of pregnancy [123].

Stimulation with cytokines causes naive NK cells transformation to effective killers, which requires rapid synthesis, trafficking and storage of large amounts of perforin and granzyme molecules [124]. NK cell killing of target cells is a complex, multi-stage process that is mediated by direct secretion of lytic granules at the immunological synapse. This process could be divided into four stages; first an immunological synapse forms at the point of contact with the target cells, where there is rearrangement of the actin cytoskeleton. Then, the microtubule organizing center (MTOC) of NK cells and the secretory lysosomes are polarized towards the lytic synapse. Subsequently, the secretory lysosomes settle in the plasma membrane before fusing with it releasing their toxic contents. The exocytosis of secretory lysosomes is a highly regulated and ordered process to prevent random NK cell-mediated killing [125,126,127,128]. 

Perforin and granzyme B are lytic enzymes that induce cell death in virally infected cells or tumor cells. Perforin facilitates the entry of the granzymes into the target cell cytoplasm, where they cleave a variety of targets, such as caspases, resulting in activation of apoptosis [129,130]. Perforin is a multi-domain molecule activated by a cysteine protease, and is a pore forming cytolytic protein that is rendered inactive when it binds calreticulin and serglycin [129]. The activity of perforin is highly dependent on pH and Ca^2+^, where its activation occurs after an evident increase in cytosolic Ca^2+^ concentration, whereas the pH of the environment should be neutral at the immunological synapse [131,132,133]. However, it is inactive inside the acidic secretory granule which is a safe storage compartment for perforin. Immunoregulatory CD56^bright^ NK cells were reported to have potentially lower levels of perforin than the more mature cytotoxic CD56^dim^ NK cells [35,134,135,136]. On the other hand, the granzymes belong to a group of serine proteases that are produced as proenzymes which remain inactive inside the secretory lysosomes, but are activated via N-terminal cleavage by dipeptidyl peptidase I, also known as cathepsin C [137,138,139,140]. Granzyme B has been described as the most potent pro-apoptotic molecule, due to its ability to cleave target cell proteins. Granzyme mediated cell death is rapid and effective where target cells undergo cell death within 5–8 minutes by apoptosis after exposure to nanomolar amounts of recombinant granzyme B and perforin [124,141,142]. Additionally, NK cells express another pore-forming molecule, granulysin, that is related to a family of saposin-like proteins [143]. Granulysin is also contained within cytolytic granules and causes target cell apoptosis in a perforin dependent manner, similar to granzymes [144,145]. These molecules appear to be active against bacteria, fungi, viruses, and tumor cells leading to membrane disruption, and eventually cell lysis [146]. Therefore, they are key players in the cytotoxicity process mediated by NK cells. 

## 4. Concluding Remarks

ILCs are innate cells that receive tissue-specific signals from the surrounding micro-environment and accordingly respond by releasing various cytokines and chemokines, as well as lytic enzymes and proteins. Consequently, they regulate tissue homeostasis, inflammation, toxicity and/or repair of various tissues including the skin, lungs, intestine, and lymphoid organs. In this review, the four subclasses of ILCs have been briefly discussed as vital mediators of inflammation. Table 1 is a summary of surface markers, transcription factors, released cytokines, as well as the functions of each of these ILC groups in physiological and pathological conditions. Considerable research must be carried out to further investigate and explore the role of these immune cells in multiple disorders.

## Figures and Tables

**Table 1:** Brief description of the different groups of Innate Lymphoid Cells (ILCs). This table summarizes the characteristics of each of the ILC groups such as surface markers as well as transcription factors. Additionally, this table recaps the protective and the pathological roles of ILCs in various conditions and diseases. Abbreviations: CCR: C-C chemokine receptor, CD: Cluster of Differentiation, COPD: Chronic Obstructive Pulmonary Disease, CRTH2: Chemoattractant receptor-homologous molecule, Eomes: eomesodermin, GATA3: GATA-binding protein 3, GM-CSF: Granulocyte macrophage colony-stimulating factor, GvHD: Graft versus host disease, IBD: Inflammatory bowel disease, IFN-γ: Interferon-gamma, IL: Interleukin, ILC: Innate lymphoid cell, LIF: Leukemia inhibitory factor, Lin: Lineage marker, NCR: Natural cytotoxicity receptor, NK: Natural Killer Cell, NKG2D: Natural Killer Group 2D, RANKL: Receptor Activator of nuclear factor kappa-B ligand, ROR: Retinoic acid receptor-related orphan receptor, ST2: component of the IL-33 receptor, T-bet: T-box transcription factor-Tbx21, TNF α: Tumor necrosis factor alpha.

ILC Group	Characteristics (Cell Surface Markers & Transcription Factors)	Cytokines Released	Role
ILC1s (NK cells, helper ILC1s)	NK cells: Lin^−^, NCR^+^, NKG2D^+^, Eomes^+^, T-bet^+^, ILC1s: CD117^−^, T-bet^+^, Eomes^−^	IFN-γ, TNF-α	NK cells: Antiviral & antitumor activity ILC1s: Anti-bacterial, IBD, Crohn’s disease
ILC2s	Lin^−^, CD45^+^, RORα^+^, Gata-3^+^, IL-7Rα^+^, ST2^+^, CRTH2^+^, CD161^+^	GM-CSF, IL-3, IL-4, IL-5, IL-6, IL-8, IL-9, IL-13, IL-21	Protection against helminth, tissue repair & homeostasis, contribution in lung tissue inflammation (asthma, CRS), AD
ILC3s (LTis, ILC3s)	Lin^−^, IL-7Rα^+^, RORγt^+^, CCR6^+^, CD117^+^, CD40L^+^, RANKL^+^, NCR^+/−^	GM-CSF, LIF, IL-17, IL-22	LTis: lymphoid tissue organogenesis ILC3s: Colitis, maintenance of commensal bacterial species, immunity against enteric bacteria, multiple sclerosis, COPD, defense against GvHD, psoriasis
ILC4s (NK17/NK1 cells)	Lin^−^, CD3^−^, CD19^−^, CD14^−^, CD56^+^, IL-7Rα^−^, CCR4^+^, IL-2R^+^, NCR^+^, NKG2D^+^, T-bet^+^, RORγt^+^	IL-17, IFN-γ	Multiple sclerosis, psoriasis

## Abbreviations

aLP	α-lymphoid precursor
AD	Atopic Dermatitis
BAFF	B-cell activating factor (BAFF)
CCR/L	Chemokine receptor/ligand
CLP	Common lymphoid progenitor
CRS	Chronic rhinosinusitis
CRTH2	Chemoattractant receptor-homologous molecule
CSF	Cerebrospinal fluid
Eomes	Eomesodermin
FLT3	FMS-related tyrosine kinase 3
GATA3	GATA-binding protein 3
GM-CSF	Granulocyte macrophage colony-stimulating factor
Id2	DNA-binding protein inhibitor
IFN	Interferon
I_H_	Innate helper
IL	Interleukin
ILCs	Innate lymphoid cells
KIR	Killer immunoglobulin-like receptor
LIF	Leukaemia inhibitory factor
Lin	Lineage marker-negative
LN	Lymph node
LTi	Lymphoid tissue inducer
MDC	Macrophage derived chemokine
MS	Multiple sclerosis
NCR	Natural cytotoxicity receptor
NHC	Natural helper cell
NK	Natural killer
PGD2	Prostaglandin D2
PLZF	Promyeloid leukaemia zinc finger
RAG	Recombination activating gene
RANKL	Receptor activator of nuclear factor kappa- B ligand
ROR	Retinoic acid receptor-related orphan receptor
T-bet	T-box transcription factor-Tbx21
T_H_	T helper cell
TNF	Tumour necrosis factor
TSLP	Thymic stromal lymphopoietin
